# Metabolic engineering of *Synechocystis* sp. PCC 6803 for the improved production of phenylpropanoids

**DOI:** 10.1186/s12934-024-02330-3

**Published:** 2024-02-18

**Authors:** Kateryna Kukil, Pia Lindberg

**Affiliations:** Microbial Chemistry, Department of Chemistry - Ångström, Uppsala University, Box 523, SE 751 20 Uppsala, Sweden

**Keywords:** *Synechocystis* sp. PCC 6803, Phenylalanine ammonia lyase, *Trans*-cinnamic acid, *p-*coumaric acid, Phenylpropanoids, 4-hydroxyphenylpyruvate dioxygenase

## Abstract

**Background:**

Phenylpropanoids are a large group of plant secondary metabolites with various biological functions, derived from aromatic amino acids. Cyanobacteria are promising host organisms for sustainable production of plant phenylpropanoids. We have previously engineered *Synechocystis* sp. PCC 6803 to produce *trans*-cinnamic acid (*t*CA) and *p-*coumaric acid (*p*Cou), the first intermediates of phenylpropanoid pathway, by overexpression of phenylalanine- and tyrosine ammonia lyases. In this study, we aimed to enhance the production of the target compounds *t*CA and *p*Cou in *Synechocystis*.

**Results:**

We eliminated the 4-hydroxyphenylpyruvate dioxygenase (HPPD) activity, which is a competing pathway consuming tyrosine and, possibly, phenylalanine for tocopherol synthesis. Moreover, several genes of the terminal steps of the shikimate pathway were overexpressed alone or in operons, such as aromatic transaminases, feedback insensitive cyclohexadienyl dehydrogenase (TyrC) from *Zymomonas mobilis* and the chorismate mutase (CM) domain of the fused chorismate mutase/prephenate dehydratase enzyme from *Escherichia coli.* The obtained engineered strains demonstrated nearly 1.5 times enhanced *t*CA and *p*Cou production when HPPD was knocked out compared to the parental production strains, accumulating 138 ± 3.5 mg L^−1^ of *t*CA and 72.3 ± 10.3 mg L^−1^ of *p*Cou after seven days of photoautotrophic growth. However, there was no further improvement when any of the pathway genes were overexpressed. Finally, we used previously obtained AtPRM8 and TsPRM8 *Synechocystis* strains with deregulated shikimate pathway as a background for the overexpression of synthetic constructs with *ppd* knockout.

**Conclusions:**

HPPD elimination enhances the *t*CA and *p*Cou productivity to a similar extent. The use of PRM8 based strains as a background for overexpression of synthetic constructs, however, did not promote *t*CA and *p*Cou titers, which indicates a tight regulation of the terminal steps of phenylalanine and tyrosine synthesis. This work contributes to establishing cyanobacteria as hosts for phenylpropanoid production.

## Introduction

Cyanobacteria as microbial cell factories possess high potential for sustainable production of various industrially relevant chemicals by direct carbon capture and conversion. High production of a target compound requires a significant redirection of metabolic flux for its synthesis, which might be challenging to achieve for the products of interest that are derived from secondary metabolism [6]. For instance, the shikimate pathway for biosynthesis of aromatic amino acids is not only involved in synthesis of various highly valuable aromatic compounds, but also formation of plastoquinone, which plays an essential role in photosynthesis, is dependent on products of this pathway. Thereby, redirecting carbon flux into the shikimate pathway could potentially lead to high productivity of native and non-native aromatic metabolites, but also to substantial alterations in the native metabolism of the cell. Only a few studies are available in the literature regarding the function and regulation of this pathway in cyanobacteria, and this limitation in knowledge makes rational metabolic engineering of the pathway for production of high value chemicals challenging.

**Fig. 1:**
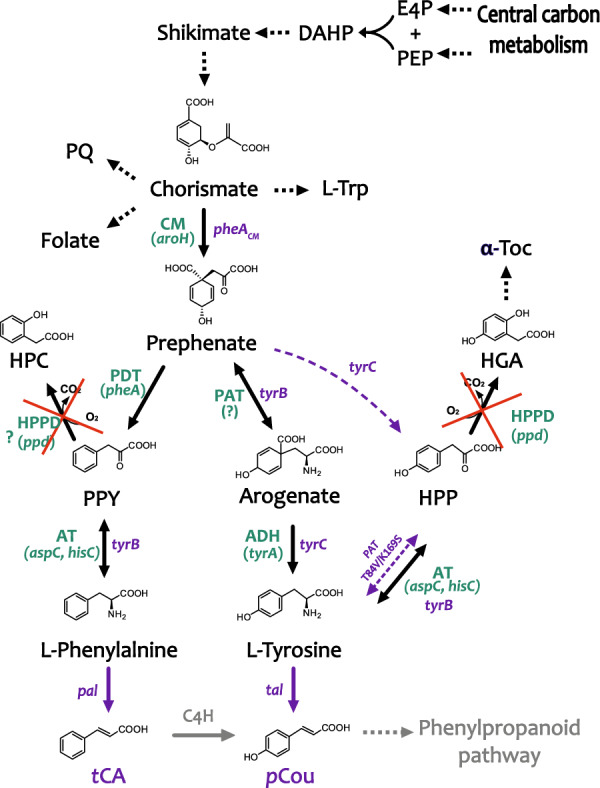
Schematic overview of the phenylalanine and tyrosine biosynthesis pathway in *Synechocystis*. The native enzymes annotated in KEGG are shown in green color with corresponding genes in brackets; the genes overexpressed in this study are indicated in purple color. Question marks corresponds to genes and enzymes that have not been identified yet. Abbreviations: ADH – arogenate dehydrogenase; AT—aminotransferase; CM-chorismate mutase; DAHP—3-deoxy-D-arabino-heptulosonate-7-phosphate; E4P—erythrose-4-phosphate; HGA—homogentisate; HPC—hydroxyphenyl acetate; HPP—4-hydroxyphenylpyruvate; HPPD—4-hydroxyphenylpyruvate dioxygenase; L-Trp—L-tryptophan; PAT – prephenate aminotransferase; PDT – prephenate dehydratase; PEP—phosphoenolpyruvate; PPY -phenylpyruvate; *p*Cou—*para*-coumaric acid; PQ—plastoquinone; *t*CA—*trans*-cinnamic acid; Tyr—tyrosine; α-Toc—α-Tocopherols

The shikimate pathway is the common metabolic route for synthesis of the three aromatic amino acids L-phenylalanine (Phe), L-tyrosine (Tyr), and L-tryptophan (Trp), as well as of folic acid and vitamins K and E. The pathway starts from condensing erythrose-4-phospate (E4P) and phosphoenolpyruvate (PEP) to yield 3-deoxy-d-arabinoheptulo-sonate 7-phosphate (DAHP) catalyzed by DAHP synthase (Fig. 1). Through six consecutive reactions, DAHP undergoes cyclisation to form chorismate. Chorismate is a last shared precursor in synthesis of the three aromatic amino acids as well as other pathway products, which makes chorismate a main metabolic hub before branching of the shikimate pathway.

The Phe and Tyr terminal branch starts with the enzyme chorismate mutase (CM) that catalyzes chorismate isomerization to prephenate, which is a common precursor for both amino acids. Phe is formed via dehydration of prephenate to phenylpyruvate (PPY) and its subsequent transamination. Tyr synthesis in cyanobacteria is suggested to occur via the arogenate route, where prephenate is directly transaminated to arogenate and converted by arogenate dehydrogenase to Tyr. Arogenate dehydratase activity was not found in multiple cyanobacterial strains tested, suggesting that no Phe is formed from arogenate [7]. In some organisms, such as *Zymomonas mobilis,* a single enzyme, cyclohexadienyl dehydrogenase (TyrC), can use both arogenate and prephenate as substrates for synthesis of Tyr and 4-hydroxyphenylpyruvate (HPP), respectively [8].

As an alternative to the arogenate route, tyrosine can be formed via HPP, which is common among microorganisms including *E. coli*. However, in cyanobacteria based on available data in KEGG (https://www.kegg.jp)*,* the HPP intermediate seems to be involved in tyrosine catabolism, where it may undergo oxidation to homogentisate (HGA), catalyzed by the enzyme 4-hydroxyphenylpyruvate dioxygenase (HPPD) (Fig. 1). In plants, HGA is a common precursor of plastoquinone and vitamin E (tocopherols and tocotrienols) synthesis. Unlike plants, *Synechocystis* sp. PCC 6803 (hereafter *Synechocystis*) does not require HPPD activity for plastoquinone synthesis [9]. A strain with a deletion of the gene encoding HPPD showed no growth impairment or change of photosynthetic pigments, but was deficient in tocopherols [10]. The substrate selectivity of HPPD varies among different organisms, demonstrating broad substrate preference in mammalian enzymes [11, 12] or absolute specificity for HPP as a substrate in *Streptomyces avermitilis* [13]. It is thereby not possible to suggest the potential substrate preference of the *Synechocystis* HPPD enzyme, whether it uses only HPP or possibly PPY, or other aromatic and non-aromatic keto-acids as substrate (Fig. 1).

Interestingly, the prephenate aminotransferase (PPA-AT) catalyzing the transamination of prephenate to arogenate has not been identified yet in cyanobacteria, whereas in plants it was discovered not so long ago [14]. In *Synechocystis,* two branched-chain aminotransferases encoded by *slr0032* (UniprotKB P54691) and *sll0402* (*aspC*, NCBI accession no WP_010873487.1), annotated as aspartate aminotransferases, were purified as recombinant proteins and demonstrated very little PPA-AT activity, while some aromatic aminotransferase activity was detected using phenylpyruvate and HPP as keto-acids and glutamate as aminodonor [15, 16]. Another gene, *sll1958* (*hisC*, UniprotKB P73807) encoding a putative histidinol-phosphate aminotransferase, is annotated in KEGG to be involved in transamination of aromatic amino acids. Altogether, the scarcity of information available in the literature and databases makes it difficult to predict how the final transamination steps of Phe and Tyr synthesis might be regulated in cyanobacteria.

We have previously engineered the model cyanobacterium *Synechocystis* to produce *trans*-cinnamic (*t*CA) and *p*-coumaric acids (*p*Cou) by overexpression of phenylalanine ammonia lyase (PAL) and tyrosine ammonia lyase (TAL), respectively [17]. Both enzymes perform a non-oxidative deamination of the corresponding amino acid, forming carboxylic acids that are the starting metabolites of the plant phenylpropanoid pathway (Fig. 1). In this work, we aimed to enhance the production of *t*CA and *p*Cou compounds in the respective *Synechocystis* strains, AtC and TsC. As the first target, we knocked out the *ppd* gene encoding the enzyme HPPD (Fig. 1). By abolishing the activity of this enzyme, we hypothesize that the availability of amino acids and precursors may increase inside the cells, which could lead to higher *p*Cou and possibly *t*CA productivity. We also tested the expression of several heterologous aromatic transaminases, since the function of this metabolic step of the shikimate pathway in cyanobacteria is still unclear. It is plausible, that the heterologous transaminases, insensitive to native regulation mechanisms, will facilitate flux through the pathway. In order to enhance the *p*Cou productivity, we overexpressed the cyclohexadienyl dehydrogenase (TyrC) from *Zymomonas mobilis*, encoded by the *tyrC* gene. Together with *ppd* knock-out and heterologous transaminases, the created metabolic route from prephenate into HPP and Tyr might be favorable for *p*Cou overproduction. Finally, in order to increase the carbon flux from chorismate to the Phe and Tyr terminal branches, we overexpressed the CM domain of the fused chorismate mutase/prephenate dehydratase enzyme from *E. coli.* It was demonstrated previously, that when the CM domain is expressed alone, it retains catalytic activity and becomes insensitive to L-Phe inhibition [18].

## Results and discussion

### Strain engineering

In this study, we aimed to enhance the *tCA* and *p*Cou production in *Synechocystis* by overexpressing several heterologous enzymes from the terminal steps of the shikimate pathway. The *ppd* locus (*slr0090*) encoding HPPD was used as the chromosomal integration site. In cyanobacteria, HGA formed by the conversion of HPP by HPPD enzyme is a precursor of tocopherols, which makes it a competing pathway to the *t*CA and *p*Cou biosynthesis. Therefore, an integration plasmid, pPPD was constructed to disrupt the *ppd* gene by insertion of an antibiotic cassette (see Table 1).

**Table 1:**
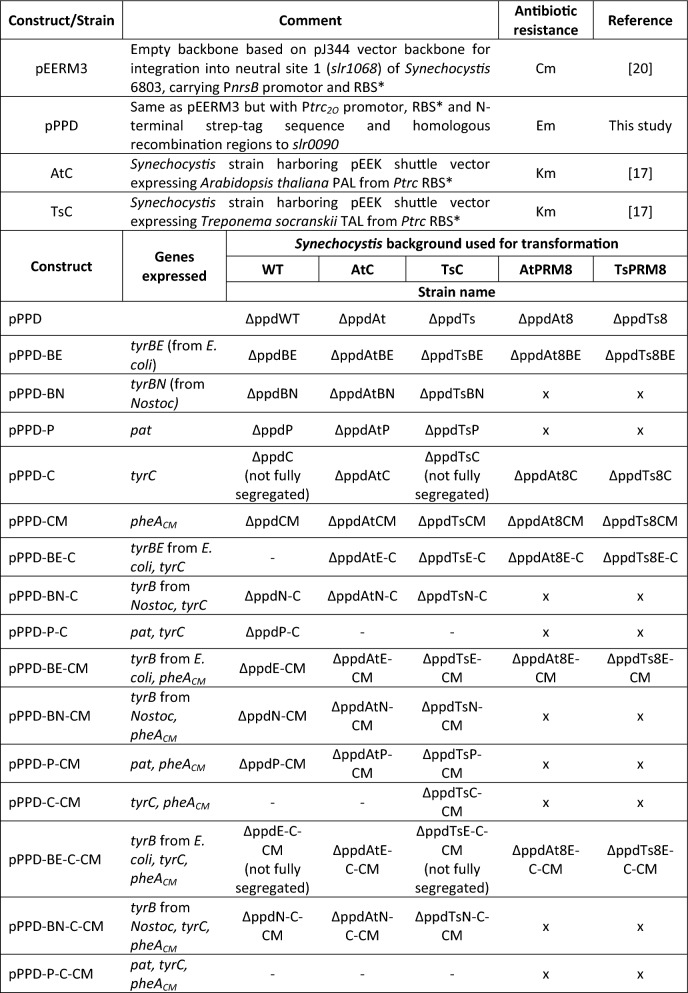
List of plasmids and strains used in this study [17, 20]

‘-’ indicates that no colonies appeared after transformation. ‘x’ represent combinations not included in the study.

To test the effect of overexpression of aromatic transaminases in *Synechocystis,* three candidate enzymes were selected (Fig. 1). The first was TyrB, a pyridoxal 5′-phosphate dependent tyrosine aminotransferase from *E. coli* encoded by *tyrB*, that has been shown to catalyze reversible transamination of phenylalanine, tyrosine, tryptophan, aspartate, glutamate, and the corresponding keto-acids [19]. Interestingly, this *E. coli* tyrosine aminotransferase in protein sequences alignment to available databases, shows sequence similarity to a few hypothetical transaminases in the genomes of newly isolated cyanobacterial species. As a second candidate for overexpression in *Synechocystis,* we selected one of these hypothetical aromatic amino acid transaminases, encoded by a similar *tyrB* gene, from the recently isolated symbiotic *Nostoc* sp. 3335mG (hereafter *Nostoc*), which may possess interesting enzymatic activity. The third enzyme candidate was a modified plant PPA-AT (gene name abbreviated as *pat* in this study) from *Arabidopsis thaliana* with two amino acid substitutions, T84V and K169V. These mutations were reported to result in complete loss of the native PPA-AT and aspartate aminotransferase activity of this enzyme, but instead introduced a new activity that efficiently transaminated HPP into Tyr using alanine and tryptophan as aminodonors [16].

In order to enhance the carbon flux towards Tyr and consequently *p*Cou production, we also overexpressed a feedback-insensitive cyclohexadienyl dehydrogenase, TyrC from *Zymomonas mobilis*, which is able to catalyze both reactions of converting arogenate to Tyr and prephenate into HPP [8] (Fig. 1). In combination with each of the transaminases, the created metabolic route from prephenate into HPP might be favorable for Tyr production. Finally, overexpression of the CM domain (*pheA*_*CM*_) of the fused chorismate mutase/prephenate dehydratase enzyme from *E. coli*, insensitive to Phe inhibition, is expected to increase the carbon flux towards the pathway end products [21] (Fig. 1). All the genes were cloned into the pPPD vector individually or organized in operons, with expression driven by the strong constitutive promotor P*trc2O* [22]. The resulting constructs were transformed into three *Synechocystis* strains: WT, AtC, TsC [17]. The AtC and TsC strains are used as control strains for production comparison. All created plasmids and strains are summarized in Table 1.

Interestingly, for some constructs, we were unable to obtain colonies after transformation. For instance, construct pPPD-BE–C, expressing *tyrB* from *E. coli* and *Z. mobilis tyrC* in an operon, was not possible to generate in the WT background, but for PAL and TAL expressing *Synechocystis* strains full segregation was successfully achieved. The construct pPPD-P–C-CM, generated to express PAA-AT, TyrC and CM from an operon, could not be transformed into any of the *Synechocystis* strains used, suggesting that the combined expression of the enzymes has a detrimental effect on the cell. Additionally, we noticed that for some constructs (pPPD-C, pPPD-CM) the full segregation was possibly combined with compensatory mutation(s), since the color of partially segregated colonies was changed from pale to healthy bright green when full segregation was achieved. In the cases of ΔppdC, ΔppdTsC, ΔppdE-C-CM and ΔppdTsE-C-CM completely segregated strains were not obtained.

### Comparative production of tCA and pCou by engineered strains

Engineered *Synechocystis* strains were cultivated under constant light intensity for seven days and sampled on days four and seven for LC–MS analysis to detect Phe, Tyr, *t*CA and *p*Cou in the growth medium. No Phe or Tyr was detected in the culture medium of strains obtained with the WT background. It appears that the knockout of *ppd* and introduction of the pathway genes alone or in any of the obtained combinations, were not sufficient to promote secretion of Phe or Tyr from the cell. The presence of a heterologous sink may be crucial for efficient consumption of the central metabolites Phe and Tyr.

**Fig. 2:**
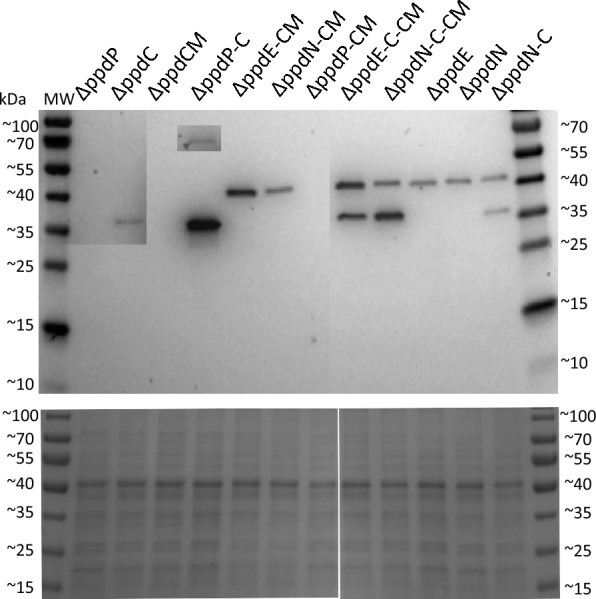
Expression of proteins in WT-based engineered *Synechocystis* strains. Upper panel displays the Western Blot using anti-Strep antibody with a total duration of exposure of 600 s and 1000 s (inset darker areas, columns ΔppdP, ΔppdC, and top band in ΔppdP-C); bottom panel shows the SDS-PAGE with 9 μg of protein crude extract. PPA-AT corresponds to the band size of ~ 51 kDa, TyrBE ~ 43,5 kDa, TyrBN ~ 41,6 kDa and TyrC to 32,1 kDa respectively

Western blot analysis (Fig. 2) was performed on WT-based strains, since Western blots of extracts from the AtC and TsC strains exhibited extra bands of smaller molecular sizes [23], overlapping and interfering with detection of other strep-tagged proteins. Interestingly, the results showed that the protein level of TyrC when expressed alone in the not fully segregated strain ΔppdC is considerably lower than when expressed as a second gene in an operon. No expression of the plant PPA-AT was detected at tested conditions in strains ΔppdP and ΔppdP-CM, whereas in the ΔppdP-C strain, the PAA-AT was barely detectable while TyrC showed the thickest band. Plausibly, the activity of the upstream transaminase enzymes when combined with TyrC decreased the possible burden on the cell such as depletion of prephenate pool for phenylalanine synthesis caused by TyrC activity. The CM domain tagged with Strep-tag was not detectable by Western blot possibly due to its smaller molecular size, however in the screening tests in *E. coli* the corresponding band of ~ 12.5 kDa was present (data not shown).

**Fig. 3:**
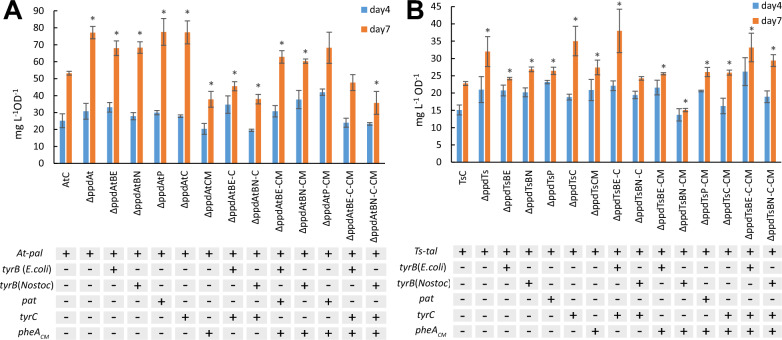
Specific production titer of *t*CA (**A**) and *p*Cou (**B**) of *Synechocystis* strains during photoautotrophic growth. Values are the means of three biological replicates and two technical replicates, error bars represent the standard deviation. Statistically significant differences were examined by Student’s *t*-test and are represented by an asterisk (*P < 0.05)

**Fig. 4:**
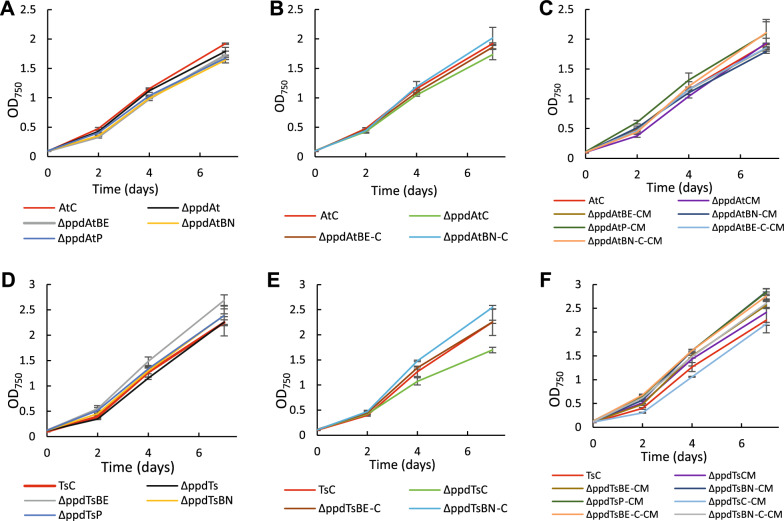
Growth of *t*CA (**A**, **B**, **C**) and *p*Cou (**D**, **E**, **F**) producing *Synechocystis* strains during seven days of experiment. Results are the mean of three biological replicates, error bars represent standard deviation

The specific production titer of accumulated *t*CA and *p*Cou in the growth medium during photoautotrophic growth is presented in Fig. 3. The *t*CA production of the ΔppdAt strain was nearly 1.5 times higher than that of the parental strain AtC accumulating 138 ± 3.5 mg L^−1^ of *t*CA. The overexpression of transaminases individually as well as *tyrC* gene alone showed no improvement compared to ΔppdAt, suggesting that the *ppd* deletion was crucial for the titer increase. The growth of all AtC-based engineered strains was similar to the control strain AtC (Fig. 4A–C).

The productivity of *p*Cou in most engineered strains expressing TsTAL together with the *ppd* deletion was similarly increased, whereas the titer of ΔppdTsC strain increased nearly 1.5 times compared to the control strain TsC, reaching 72.3 ± 10.3 mg L^−1^ after seven days, (Fig. 3B). Except for ΔppdTsBN-CM strain, there were no significant difference in *p*Cou titer among the strains with *ppd* knocked out. The obtained results also demonstrate little increase of *p*Cou specific production from day four to seven, suggesting that Tyr synthesis is less coupled to growth phase of the cells than Phe. In case of *t*CA, production normalized per OD_750_ at day seven doubled to that of day four in some strains. It is plausible, that at early growth stages Phe would be incorporated into biomass, whereas when cells divide less frequently, more Phe becomes available for secondary metabolism synthesis. The growth of the overexpression strains was slightly better than the corresponding control strain TsC (Fig. 4 D, E, F), with the exception of ΔppdTsC and ΔppdTsC-CM strains, likely due to detrimental effects of TyrC gene overexpression, which also prevented full segregation in these strains. As mentioned above, possibly, the expression of TyrC negatively affects the cell growth due to depletion of the prephenate pool for phenylalanine synthesis, whereas when combined with any of the aminotransferase enzymes, the negative effect is relieved.

**Fig. 5:**
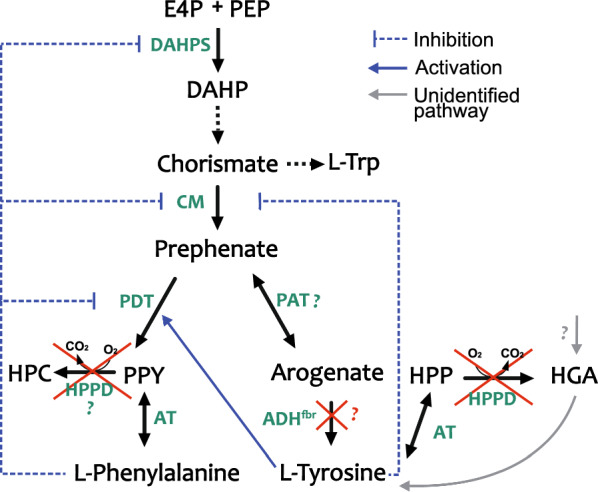
Enzyme regulation of Phe and Tyr biosynthesis possibly existing in *Synechocystis*. Summarized and adapted from references [1–4] and also based on results in this study. Abbreviations: CM chorismate synthase; DAHP, 3-deoxy-D-arabinoheptulosonate 7-phosphate; DAHPS, DAHP synthase; E4P, erythrose-4-phosphate; HGA homogentisate; HPC hydroxyphenyl acetate; HPP, 4-hyroxyphenylpyruvate; HPPD, 4-hydroxyphenylpyruvate dioxygenase; PAT prephenate aminotransferase PDT prephenate dehydratase; PEP, phosphoenolpyruvate; PPY, phenylpyruvate

Overexpression of the CM domain insensitive to Phe inhibition (ΔppdAtCM, Fig. 3A) led to a decrease of the *t*CA synthesis compared to both AtC and ΔppdAt strains, whereas the *p*Cou titer in strain ΔppdTsCM (Fig. 3B) was at the same level as the ΔppdTs control. An increase of intracellular prephenate availability theoretically should enhance the flux of precursors to Phe and Tyr. The *p*Cou production and consequently Tyr synthesis, was not affected by CM overexpression. Previously, it was suggested that the native prephenate dehydratase, encoded by *pheA,* is subject to feedback inhibition by Phe in the closely related strain *Synechocystis* sp. 29108 [2]. The overexpression of transaminases in an operon with CM restored the titer of *t*CA to control AtC strain levels (Fig. 3A, strains ΔppdAtBE-CM, ΔppdAtBN-CM and ΔppdAtP-CM). Since TyrB from *E. coli* has a broad aromatic substrate specificity, and although the substrate preference of TyrB from *Nostoc* is unknown, it is possible that the heterologous transaminase activity redirects the carbon flux into arogenate and Tyr (see Fig. 1) and thus relieves the inhibition of PheA by Phe. Possible regulation mechanisms involved in Tyr and Phe synthesis in *Synechocystis* are summarized in Fig. 5.

The overexpression of TyrC alone, which acts only on prephenate and arogenate as substrates resulted in similar *t*CA production titers as in the ΔppdAt strain, whereas the expression of TyrB transaminases in operon with TyrC decreased productivity to AtC strain levels (Fig. 3A), possibly because of a stronger partitioning of substrate towards Tyr. Integration of the TyrC overexpression cassette in WT and TsC never reached complete segregation. The double construct of *tyrC* and *pheA*_*CM*_ in an operon were not transformable in WT or AtC, suggesting that expression of these genes together is only viable when combined with TAL overexpression, although it did not enhance the *p*Cou titer in ΔppdTsC-CM strain.

The intracellular tyrosine concentration might be the crucial key to this regulatory puzzle. The study by Hall and Jensen [2] suggested that Tyr acts as an activator of PheA (Fig. 5), and the activation effects of Tyr is as strong as the inhibiting effect of Phe. Possibly, the pattern of coupled Tyr and Phe biosynthesis, where enzyme activity of one branch can be affected by the product of another, ensures the required ratio of Phe/Tyr synthesis for the cellular needs [24]. Indeed, it has been shown previously that the addition of a degradation tag to PheA in an aromatic amino acid overproducer strain expressing feedback resistant *aroG* and *TyrA* from *E. coli* resulted not only in decrease of Phe, but also in Tyr [25]. In our case, *tyrC* overexpression in combination with *pheA*_*CM*_ and transaminases would enhance Tyr biosynthesis, leading to PheA activation, thus redirecting the flux away towards Phe synthesis and thereby no increase of *p*Cou titer is observed. In case of the PAL overexpression strains, ΔppdAtE-C-CM and ΔppdAtN-C-CM had no improvement of *t*CA titer with respect to control AtC, whereas strains ΔppdAtE-C and ΔppdAtN-C produced less than the AtC control strain. In analogy with the above reasoning, tyrosine activation of the Phe branch would be caused by initial Tyr accumulation but will not result in a net accumulation of Phe, as the upstream pathway will then be downregulated to maintain a balanced Phe/Tyr ratio.

Similarly as observed previously [17, 26], the effect of the heterologous sink created by PAL overexpression results in slower growth, possibly due to decreased intracellular Phe concentration. This could further indicate that only the elevated Phe levels are controlling the flux into shikimate pathway via DAHP synthase inhibition, whereas the cell response to low Phe levels is slowing down the growth and biomass formation rather than compensatory pathway upregulation due to consumption of Phe by PAL. This is further supported by the observation that overexpression of feedback resistant DAHP synthase and TyrA from *E.coli* [25] led to Phe and Tyr accumulation in the media, but lower biomass accumulation, while improved growth rate was obtained when the natural mutagenesis was applied [26, 27].

The strong re-routing of Phe to *t*CA may have other detrimental effects on the cell, such as in response to high concentrations of *t*CA, or effects on regulation of other reactions and related metabolites. With regard to effect of high *t*CA concentration, in our previous work supplementation of AtC culture with Phe during photoautotrophic growth resulted in the Phe conversion into *t*CA with the tCA concentration above 0.5 g/L and the growth of the culture was not affected [26]. Nevertheless, since all the reactions involved Tyr and Phe metabolism in cyanobacteria are not characterized, potential effects are difficult to predict.

Undoubtedly, the regulation of Phe and Tyr synthesis is tight and complex, and their intracellular concentrations is probably maintained in a dynamic equilibrium. Metabolic flux analysis would be important to identify the partitioning of precursors into the Phe or Tyr biosynthetic branch. Jensen and Hall suggested that cyanobacterial control of the aromatic amino acids pathway is endo-oriented, where the pathway regulation is tuned to the endogenous formation of the initial pathway substrates, and thereby the earliest enzyme controlling the flow has the major role in the end-product regulation [24]. Thus, the loss of early-pathway regulation would provide sufficient elevation of substrate levels, and eliminate the flow restrictions by terminal branch enzymes leading to extracellular accumulation of Phe and Tyr as was observed previously [25]. Similarly, in our study, there was no Phe or Tyr secretion observed with overexpression of the terminal enzymatic steps in Phe and Tyr synthesis.

### Comparative production of tCA and pCou by engineered strains in PRM8 background

As the next step, we aimed to combine obtained strains with the overexpression of a feedback resistant version of DAHP synthase, the first enzyme in the shikimate pathway, in order to relieve the early-flux controlling node. Yet, the transformation of the AroG150-pEERM3 construct into any of the engineered strains as well as *Synechocystis* WT was unsuccessful, consistently resulting in false-positive colonies after several transformation trials. An overexpression of feedback resistant *aroG* from *E.coli* in *Synechocystis* has been already demonstrated previously (*aroG*L175D [27], and in [25] with no mentioning of the mutation site). The mutation aroGL150P was described to result in an activated version of DAHP synthase [5], thus it might have placed some metabolic burden to the cell leading to an unsuccessful transformation in *Synechocystis*.

**Fig. 6:**
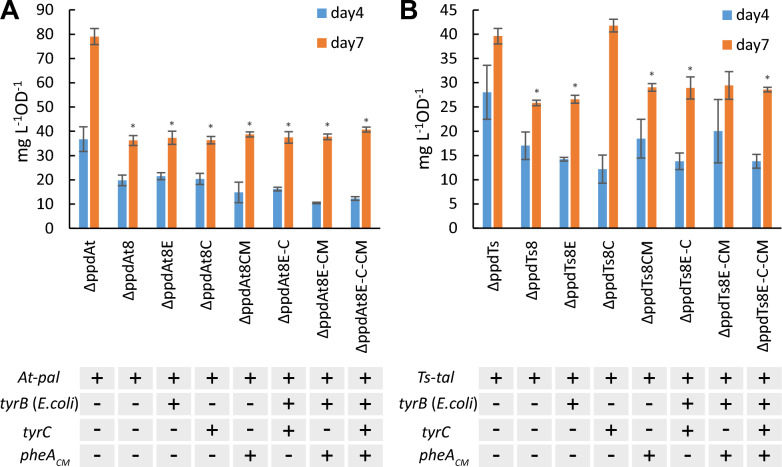
Specific production of *t*CA (**A**) and *p*Cou (**B**) during photoautotrophic growth of *Synechocystis* PRM8-based strains. Values are the means of three biological replicates and two technical replicates, error bars represent the standard deviation. Statistically significant differences were examined by Student’s *t*-test and are represented by an asterisk (*P < 0.05)

**Fig. 7:**
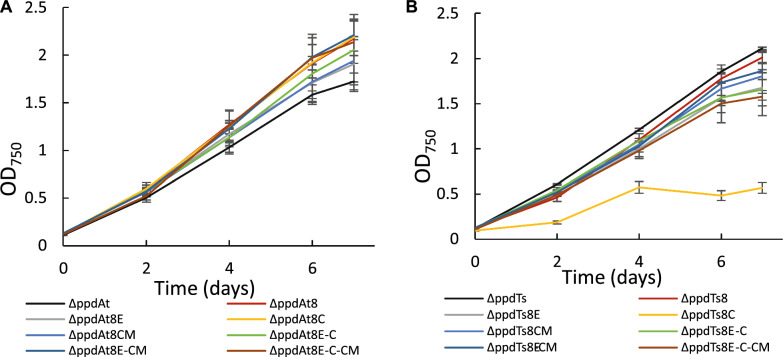
Growth of At8 (**A**) and Ts8 (**B**)-based engineered strains of *Synechocystis* during seven days of experiment. Results are the mean of three biological replicates, error bars represent standard deviation

As an alternative approach, we used PRM8 (Phenylalanine Resistant Mutant #8), obtained from previous study [26], as a background strain for overexpression of created genetic constructs. This metabolic mutant of *Synechocystis* was shown to have a deregulated shikimate pathway with a mutation in a native DAHP synthase resulting in accumulation of high volumetric titers of phenylalanine when cultivated in high-density cultivation conditions. Thus, we have overexpressed several constructs, including the *ppd* knockout, in PRM8 based strains AtPRM8 and TsPRM8 harboring PAL and TAL activities respectively [26] (for simplicity these strains’ names have been shortened to At8 and Ts8 in this paper). Productivity of resulting engineered strains (Table 1) was compared to the corresponding ΔppdAt or ΔppdTs control strains. The results of the photoautotrophic growth after 7 days demonstrated that the productivity of *t*CA was not improved when At8 was used as a background strain (Fig. 6A). The growth of PRM8 based engineered strains was similar or slightly better compared to the control ΔppdAt strain (Fig. 7A). In case of *p*Cou production, only the strain ΔppdTs8C reached specific productivity titer of the control strain ΔppdTs (Fig. 6B) although the growth of this strain was reduced nearly by half compared to the rest of the strains (Fig. 7B), while for the remaining strains the *p*Cou titer was lower compared to the control strain. Notably, the growth of all engineered *p*Cou-producing strains based on PRM8 was slower than of the ΔppdTs control, which is opposite for *t*CA-producing strains (Fig. 7). The *ppd* knockout seemed to cause a negative effect on growth of TsPRM8 strains in standard photoautotrophic conditions.

**Fig. 8:**
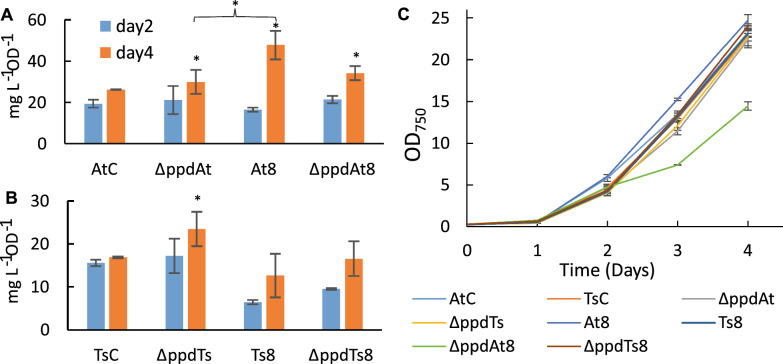
Productivity and growth of PRM8-based strains during high-density cultivation. **A** Comparison of *t*CA production between engineered and control strains. **B** Comparison of *p*Cou production between engineered and control strains. Samples were taken from the growth medium at day two and day four. The values are the means of three biological replicates and two technical replicates, error bars represent the standard deviation of three biological replicates. **C** Growth curves of tested strains during four days of cultivation. Error bars represent standard deviation of three biological replicates. Statistically significant differences were examined by Student’s *t*-test and are represented by an asterisk (*P < 0.05)

In the previous study [26], the *t*CA and *p*Cou production during shake flask cultivation experiments was decreased in PRM based strains compared to WT based AtC and TsC control strains, while high-density cultivation conditions revealed a superior productivity phenotype of PRM8 based strains. Similarly, we have performed a high-density cultivation experiment to determine the effect of *ppd* knockout on selected strains in this specific growth condition. The results presented in Fig. 8A and B showed that although *ppd* knockout increased *t*CA and *p*Cou production compared to AtC and TsC controls similarly to shake flasks experiment, when At8 was used as background, the *t*CA titer decreased with *ppd* knockout in strain ΔppdAt8. In case of *p*Cou production (Fig. 8B), ΔppdTs8 strain reached higher specific titer than Ts8, however the productivity of Ts8 strain resulted to be lower than previously described [26].

Thus, the usage of PRM8 as a background strain did not enhance *t*CA and *p*Cou titer even when combined with *ppd* knockout, as the improvement of productivity with deleted HPPD activity was only observed in WT based strains, which in summary demonstrates the complexity of shikimate pathway regulation.

### Phenotypic effect of TAL overexpression

**Fig. 9:**
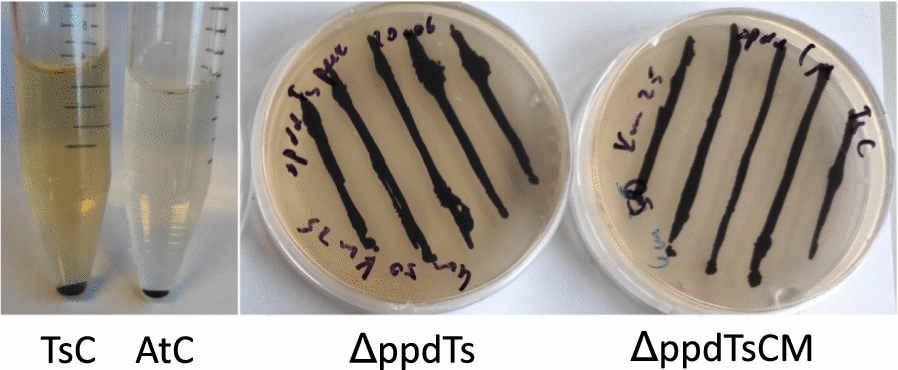
Appearance of brown color in growth medium of *Synechocystis.* Left panel: Stationary culture (two weeks of cultivation). Culture medium of AtC strain is shown for comparison. Right panel: Agar plates after incubation for several weeks

The expression of heterologous TAL in the WT or Δ*ppd Synechocystis* background was coinciding with the appearance of brown culture supernatant, which we have observed previously in experiments with TsC strain expressing TAL (K. Kukil, unpublished data) (Fig. 9). Similar evidence was reported for the overexpression of HPPD from *A. thaliana* in *Synechocystis*, and overexpression of α-ketoisocaproate dioxygenase from *Rattus norvegicus*, which possess the HPPD activity [10, 28]. The brown coloration is likely due to the formation of pyomelanin, a melanin-like pigment, a reaction product of HGA oxidation and polymerization [29]. Unlike in previous studies where the co-occurrence of the brown coloration reported is due to extra HPPD activity and thus increased HGA accumulation, in case of TsC strain, the TAL overexpression is supposed to increase the Tyr consumption by diverting it into the heterologous compound *p*Cou. It is likely that the direction of Tyr into tocopherol biosynthesis involves certain regulation. In the above-mentioned study on overexpression of *A. thaliana* HPPD, when HPPD was coexpressed with geranylgeranyldiphosphate hydratase (GGH) from *A. thaliana* in *Synechocystis*, the supernatant did not turn brown, but accumulation of tocochromanols was increased [10]. The availability of prenyl chain precursors thus seems to be a bottleneck for efficient utilization of HGA into vitamin E synthesis. Synthesis of HGA from HPP is a multistep exergonic reaction involving oxidative decarboxylation, phenyl ring hydroxylation and side-chain rearrangement, and this reaction is thus irreversible [30]. Possibly, the TAL expression in *Synechocystis* alters the Tyr partitioning leading to increased HGA synthesis and brown coloration of the media. Since the substrate usage of *Synechocystis* HPPD enzyme is not known, there is a possibility that HPPD apart from HPP may also use PPY or other aromatic and non-aromatic keto-acids (Fig. 1).

It is also puzzling that the brown color was present in the cultures of *Ts*TAL expressing strains where *ppd*, encoding HPPD, was deleted (Fig. 5). Nowicka and Kruk observed the incorporation of radiolabeled HGA into the Tyr pool in *Synechocystis*, suggesting the existence of an unknown pathway for conversion of aromatic compounds [4]. Moreover, it was reported recently that the deletion of arogenate dehydrogenase, encoded by *tyrA,* from the terminal branch of Tyr biosynthesis is possible [3]. Therefore, either the report on the *tyrA* knock out strain is in conflict with the supposed essentiality of this gene in cyanobacteria [31] or it leads to the possibility of existence of another route to Tyr. It is tempting to suggest that the brown coloration in Δ*ppd* strains is a result of HGA accumulation via a not yet identified route of aromatic compounds metabolism.

Altogether, *Synechocystis* cells exhibit a strong response to the overexpression of heterologous genes from the shikimate pathway, leading to either absence of colonies after transformation, or likely accumulating some compensatory mutations to reach full segregation. This demonstrates the high rigidity of the cellular metabolism of aromatic compounds, which makes it a challenging target for metabolic engineering.

## Conclusions

In this study following the aim to establish the cyanobacteria as microbial cell factories for sustainable production of phenylpropanoids, we engineered previously obtained *Synechocystis* strains AtC and TsC in order to enhance their *t*CA and *p*Cou productivity, respectively. We demonstrate that inactivation of HPPD promoted the *t*CA and *p*Cou synthesis nearly 1.5 times compared to parental strains, reaching 138 ± 3.5 mg L^−1^ of *t*CA and 72.3 ± 10.3 mg L^−1^ of *p*Cou after seven days of photoautotrophic growth. However, there was no further improvement of productivity, when HPPD knock out was combined with heterologous overexpression of genes from the terminal steps of phenylalanine and tyrosine synthesis, possibly due to tight regulation of the phenylalanine and tyrosine biosynthetic pathway. In order to resolve this issue we used PRM8 based strains for overexpression of the designed synthetic constructs. However, the resulting *t*CA and *p*Cou titers were not enhanced compared to WT background strains.

A comprehensive investigation of aromatic amino acid metabolism is needed, such as metabolic flux analysis in order to explore the possible existence of alternative routes leading to tyrosine formation as well as the possibility of decoupling the tyrosine synthesis from phenylalanine, which appears to be difficult in *Synechocystis.*

## Methods

### Bacterial strains and growth conditions

*Escherichia coli* DH5α-Z1 (Invitrogen) was used for subcloning and conjugation. *E. coli* cultures were cultivated in LB medium at 37 °C and supplemented with appropriate antibiotics to the final concentrations in the medium: 50 μg·ml^−1^ kanamycin (Km) or 200 μg·ml^−1^ erythromycin (Em) (Sigma, Merck).

In this study the model cyanobacterial strain *Synechocystis* sp. PCC 6803, a glucose-tolerant unicellular strain, was used. Cultures were grown in BG11 medium [32] with respective antibiotics Km 25 μg·ml^−1^ and/or Em 25 μg·ml^−1^ at 30 °C under constant 45 µmol photons m^−2^ s^−1^ light. The optical density of *Synechocystis* cultures was measured on Varian Cary 50 BIO spectrophotometer at a wavelength of 750 nm.

### Plasmid construction for gene expression

**Fig. 10:**
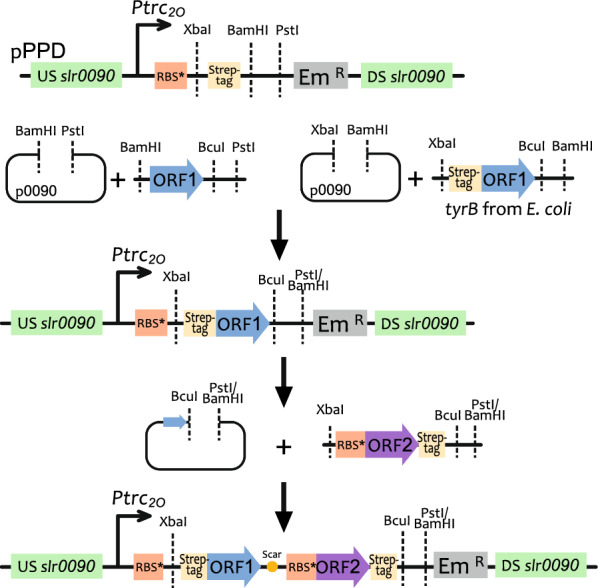
Schematic representation of vector construction. The base vector pPPD was cut with corresponding restriction site to insert ORF1 (single constructs). The addition of next ORFs was performed by cutting the new vector with BcuI and PstI (or BamHI) and the next insert with XbaI and PstI (or BamHI) and creation a BcuI/XbaI scar. DAHP synthase encoded by *aroG* was amplified from *E. coli* using *E. coli* cells as template. The gene was inserted into the integrative vector pEERM3. The expression was driven by a constitutive promotor *Ptrc2O* and synthetic ribosomal binding site RBS*, the gene sequence was followed by a flexible Gly-Ser linker and Strep-tag sequence at the C-terminal position. In order to create a feedback-resistant version of *E.coli* DAHP synthase, a Pro150Leu substitution [5] was introduced by mutagenesis PCR, creating the construct AroG150-pEERM3

In this study, all overexpressed genes were inserted in the *ppd* gene (*slr0090*) loci. For this, firstly, a *ppd* knock out vector, pPPD, was constructed based on the integrative vector pEERM3 [20]. Flanking regions of 1 kb length were amplified upstream and downstream of *slr0090* from *Synechocystis* genomic DNA and cloned into pEERM3. Then, the antibiotic resistance cassette was changed from original chloramphenicol to Em using the PstI and SacI restriction sites. A sequence of a strong constitutive promotor *Ptrc2O,* synthetic ribosomal binding site RBS* [22], followed by XbaI restriction site, Strep-tag sequence with glycine-serine linker at the N-terminus position and BamHI and PstI cloning sites was amplified form pEEKN vector [17] and cloned using EcoRI and PstI restriction sites creating the pPPD vector (Fig. 10).

*E. coli* genes for overexpression were amplified using the *E. coli* BL21(DE3) genomic DNA as template. All other genes were codon optimized and synthesized by GenScript. Transaminase genes *tyrB* form *Nostoc* sp. 3335mG (GenBank PXA95964.1) and the CDS of prephenate aminotransferase from *Arabidopsis thaliana* (At2g22250/NP_565529.1) with the amino acid substitutions T84V and K169V were carrying the 3’ overhang of a BcuI restriction site, and were cloned into the pPPD vector using BamHI and PstI restriction sites. The *tyrB* gene from *E. coli* already contained a PstI restriction site, therefore during gene amplification a 5’ overhang with XbaI restriction site followed by Strep-tag sequence and a 3’ overhang with BcuI restriction site were introduced. Then the amplicon was cloned into the pPPD vector using XbaI and BamHI sites. Each transaminase expressing construct was combined in an operon with the *tyrC* gene (UniprotKB-Q04983), encoding cyclohexadienyl dehydrogenase from *Zymomonas mobilis subsp. mobilis (*strain ATCC 31821/ZM4/CP4) [33] and/or *pheA*_*CM*_, encoding the chorismate mutase domain of the fused chorismate mutase/prephenate dehydratase from *E.coli* (UniProtKB- P0A9J8). The *tyrC* sequence fragment carried a 5’ overhang of XbaI site and RBS* and a 3’ overhang with a strep-tag sequence, BcuI and PstI (or BamHI in case of *tyrB* from *E. coli*) restriction sites. The fragment of 327 bp that corresponds to the chorismate mutase domain (residues 1 to 109) of CM/PDH from *E. coli,* in a same manner as *tyrC* carried a 5’ overhang of XbaI site and RBS* sequence and a 3’ overhang with a strep-tag sequence, and BcuI and PstI (or BamHI in case of *tyrB* from *E. coli*) restriction sites. Both fragments were cloned sequentially by cutting with XbaI and PstI, while the backbone was cut with BcuI and PstI respectively, thereby creating an ACTAGA scar from the BcuI/XbaI ligation. In addition, the *tyrC* and p*heA*_*CM*_ genes were cloned singly into pPPD vector as well as two together without an upstream transaminase gene.

### Transformation of *Synechocystis*

For transformation, *Synechocystis* WT cells, PRM8 strain as well as engineered strains already possessing the Km resistance, were transformed with the series of pPPD expression vectors as described previously [22]. Colonies that appeared after 10–14 days were analyzed using PCR and restreaked on plates repeatedly or cultivated in liquid BG11 medium with appropriate antibiotics until full segregation was reached.

### Western blotting

Soluble protein fraction of *Synechocystis* cells was extracted as described previously by Ivleva and Golden 2007 [34]. Proteins were separated by SDS-PAGE, using Mini-PROTEAN TGX™ gels (Bio-Rad), and transferred to a PVDF membrane (Bio-Rad) according to standard protocols. For detection of strep-tagged proteins Anti-Strep-tag II (Abcam) antibodies were used.

### Determination of tCA and pCou by LC–MS

Determination of *t*CA and *p*Cou in the growth media was performed by LC–MS. For this 1 ml of supernatant was taken from *Synechocystis* cultures at certain days of experiment, samples were filtered through 0.2 μm pore PTFE filters (Fisherbrand) and subjected to HPLC analysis. Samples were stored at − 20 °C if not analyzed the same day.

HPLC–MS analysis was performed using an Agilent 1290 Infinity II HPLC system equipped with a 1290 Infinity II High Speed pump and a 1260 II Infinity DAD HS UV–vis detector, using an InfinityLab POROSHELL SB-120 C18 column with dimensions of 100 mm × 2.1 mm and 2.7 μm particle size. The HPLC was coupled to an InfinityLab LC/MSD equipped with an ESI ionization source. LC separation was performed using a water (A, 0.1% formic acid) and acetonitrile (B) eluent system using the method: 0–1 min 10% B; 1–10 min 10 → 90% B; 10–11 min 90% B; 11–11.1 min 90–10% B, 11.1–12 min 10% B; at a flow rate of 0.3 ml/min. The quantification of *t*CA and *p*Cou in *Synechocystis* cultures was based on a linear calibration curve from standards measured in technical triplicates. Standards for *tCA* and *p*Cou (Sigma, Merk) were prepared in BG11 medium in the range 1–200 μg·ml^−1^ and filtered before analysis.

## Data Availability

The datasets used and/or analysed during the current study are available from the corresponding author on reasonable request.

## Abbreviations

CM: Chorismate mutase
DAHP: 3-Deoxy-d-arabinoheptulo-sonate 7-phosphate synthase
E4P: Erythrose-4-phospate
HGA: Homogentisate
HPP: 4-Hydroxyphenylpyruvate
HPPD: The 4-hydroxyphenylpyruvate dioxygenase
PAL: Phenylalanine ammonia lyase
pCou: *p-*Coumaric acid
PEP: Phosphoenolpyruvate
PPA-AT: Prephenate aminotransferase
PPY: Phenylpyruvate
TAL: Tyrosine ammonia lyase
tCA: *trans*-Cinnamic acid
TyrC: Cyclohexadienyl dehydrogenase
